# Distribution Analysis of Twelve Mycotoxins in Corn and Corn-Derived Products by LC-MS/MS to Evaluate the Carry-Over Ratio during Wet-Milling

**DOI:** 10.3390/toxins10080319

**Published:** 2018-08-06

**Authors:** Juhee Park, Dong-Ho Kim, Ji-Young Moon, Jin-Ah An, Young-Woo Kim, Soo-Hyun Chung, Chan Lee

**Affiliations:** 1 Advanced Food Safety Research Group, BrainKorea21 Plus, Department of Food Science and Technology, Chung-Ang University, 4726, Seodong-daero, Anseong-si 17546, Gyeonggi-do, Korea; bjhwngml@naver.com (J.P.); wlsdk4743@naver.com (J.-A.A.); catco1120@naver.com (Y.-W.K.); 2 National Agricultural Products Quality Management Service, 141, Yongjeon-ro, Gimcheon-si 39660, Gyeongsangbuk-do, Korea; anoldmu@korea.kr (D.-H.K.); jymoon76@korea.kr (J.-Y.M.); 3 Department of Integrated Biomedical and Life Science, Korea University, Seoul 02841, Korea

**Keywords:** corn, corn by-products, wet-milling, mycotoxin, LC-MS/MS analysis

## Abstract

This study investigated the distribution of twelve mycotoxins (aflatoxins B_1_, B_2_, G_1_, and G_2_; ochratoxin A; fumonisins B_1_ and B_2_; deoxynivalenol; nivalenol; zearalenone; T-2 toxin; and HT-2 toxin) in corn and corn by-products (corn bran, cornstarch, corn gluten, corn gluten feed, corn germ, light steep water, and corn steep liquor) produced by wet-milling in Korea. Fifty-two samples were collected from three factories producing cornstarch and other corn by-products. The samples were pretreated on an immunoaffinity column (IAC), and then the levels of the 12 mycotoxins were analyzed simultaneously by liquid chromatography-coupled triple-quadrupole mass spectrometry (LC-MS/MS). *Fusarium* mycotoxins were mainly found in raw corn and corn gluten feed samples. Other mycotoxins—such as aflatoxins, ochratoxin A, and HT-2 toxin—were detected in tiny amounts below the limit of quantification (LOQ) in cornstarch, corn germ, and corn bran. Ochratoxin A and nivalenol were mainly carried over into cornstarch. Aflatoxin B_1_, deoxynivalenol, T-2 toxin, HT-2 toxin, and the fumonisins were concentrated in corn gluten feed. Zearalenone was evenly distributed in all corn by-products except cornstarch during the milling process.

## 1. Introduction

Various mycotoxins—including aflatoxins (AFs), ochratoxins (OTs), and fumonisins (FUMs)—are produced by fungal strains during the growth, harvesting, storage, and distribution of cereals such as wheat, barley, corn, and peanuts. In general, these mycotoxins are chemically and thermally stable, surviving cereal processing and various storage conditions [1]. Therefore, these mycotoxins in grains threaten human health and cause various toxic symptoms in animals, such as feed refusal, immunosuppression, and estrogenic syndrome.

AFs are the most toxic and powerful natural carcinogens among all mycotoxins [2]. They are mainly produced by *Aspergillus* species (e.g., *A. flavus* and *A. parasiticus*) through the polyketide biosynthetic pathway [3]. OTs are produced as secondary metabolites by *A. ochraceus* and by *Penicillium* species such as *P. viridicatum*. Ochratoxin A (OTA) exhibits renal toxicity, neurotoxicity, embryotoxicity, immunosuppressive properties, carcinogenicity, and teratogenicity in vivo [4]. Many *Fusarium* mycotoxins—including nivalenol (NIV), deoxynivalenol (DON), zearalenone (ZEN), fumonisins (FUMs), and T-2 toxin (T-2)—are distributed widely in cereal products, mainly wheat and maize. *Fusarium* mycotoxins can cause various diseases in humans, animals, and even plants [5]. Due to the health concerns they create for both humans and animals, mycotoxins in food products have been extensively investigated [6].

Corn can be used directly as food or can be processed into food products such as corn flakes, corn flour, corn meal, corn oil, baby food, and starch [7]. The ease of exposure to *Fusarium* mycotoxins in corn and the risks associated with them have prompted intensive studies into their occurrence in corn and corn-derived products [8]. The worldwide occurrence of and human exposure to mycotoxins including FUMs, DON, and ZEN in corn and corn-based products have been well documented and reviewed in the literature [9,10,11,12,13]. These studies have revealed high levels of contamination by these mycotoxins, especially in corn. Furthermore, there have been many reports of mycotoxin contamination and concentration in corn caused by repeated transportation and storage in conditions appropriate for fungal growth during corn milling and further processing.

The procedures of corn processing are mainly classified as wet-milling and dry-milling [14]. Dry-milling is the physical process of removing the envelope of the grain to obtain part of the endosperm, yielding products such as corn grits, germ, and flour [13,14,15]. Wet-milling is the process of immersing corn in light steep water (LSW) for 24–36 h for efficient corn decomposition in the early stage [14,16]. These milling processes lead the production of corn by-products, which contain unevenly distributed mycotoxins after fractionation. Most corn processing factories in Korea apply wet-milling to produce cornstarch and other corn by-products.

In many cases, corn is contaminated simultaneously with various mycotoxins; however, there is little information on the co-occurrence of these contaminants during the wet-milling of corn products, as most toxicity evaluations and risk assessments to date have examined single mycotoxins. Fortunately, various methods have been developed to analyze individual mycotoxins in samples that have been pretreated on immunoaffinity columns (IACs). Mycotoxins were analyzed simultaneously in processed cereal-based baby foods [17] and in feeds [18] using IAC. Nevertheless, given the wide co-occurrence of mycotoxins and the dangers they pose to humans and animals, rapid and reliable screening methods are required to identify and quantify various mycotoxins simultaneously in compliance with current and upcoming legislation [6], particularly during the wet-milling of corn.

Recently, we developed a method for the simultaneous analysis of 12 mycotoxins using liquid chromatography-coupled triple-quadrupole mass spectrometry (LC-MS/MS) [19]. When this analytical method with low LOD and LOQ values was applied, the 12 mycotoxins could be analyzed easily and simultaneously in Korean grains. The objective of the present study was to use LC-MS/MS to determine the contamination levels of the 12 mycotoxins in corn and corn by-products, and to investigate the carry-over of these mycotoxins from corn to corn-derived products. The results of these analyses will help to control the wet-milling process so that mycotoxin-free cornstarch can be produced and the mycotoxin content of corn by-products can be reduced.

## 2. Results and Discussion

### 2.1. Method Validation

The recovery ratios, LOD values, and LOQ values of the 12 mycotoxins are shown in Table 1. The recovery ratios of DON and NIV at the level of 250.0 µg/kg were 108.8 and 128.9%, respectively. AFs displayed recovery ratios between 86.9% and 112.1%. The recovery ratio of ZEN was 93.5% at 100.0 µg/kg of spiking, and 103.1% of OTA was recovered at 25.0 µg/kg. HT-2 exhibited the lowest recovery ratio (72.7%), followed by fumonisin B_2_ (FB_2_) (81.9%), at a spiked level of 500.0 µg/kg. These results were satisfactory because the most recovery ratios except for HT-2 and NIV fulfilled the guidelines of the CODEX Alimentarius and AOAC, which recommend recovery ratios of 75–120% at the level of 10–1,000 µg/kg [20]. The LOD and LOQ values were in the range of 0.1–4.8 and 0.3–16.0 μg/kg, respectively.

Other researchers also designed a method to analyze 25 mycotoxins (including AFs, FUMs, DON, ZEN, and OTA) in maize, cassava flour, and peanut cake samples. In maize samples, the LOD and LOQ values ranged from 0.1–35 and 0.3–106 μg/kg, respectively, and the recovery ratios ranged from 73 to 113% [21]. Thus, compared with our results, these LOD and LOQ values were relatively high, while the recovery range was relatively narrow. In the US, a study was conducted to detect AFs, DON, FUMs, OTA, HT-2, T-2, and ZEN simultaneously, and the recovery ratios ranged from 80 to 120% at spike levels of 1–1000 ng/g [22].

**Figure 1:**
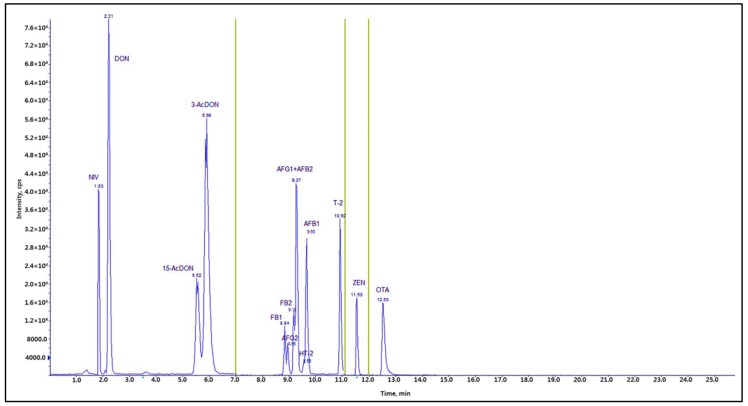
Total ion current chromatogram (TIC) of twelve mycotoxins: NIV and DON (100 ng/mL), AFB_1_ and AFG_1_ (5 ng/mL), AFB_2_ and AFG_2_ (1.24 ng/mL), FB_1_ and FB_2_ (50 ng/mL), T-2 and HT-2 (5 ng/mL), ZEN (5 ng/mL), and OTA (5 ng/mL). AFB_1_, AFB_2_, AFG_1_, AFG_2_: aflatoxins B_1_, B_2_, G_1_, and G_2_; FB_1_, FB_2_: fumonisins B_1_ and B_2_; HT-2: HT-2 toxin; T-2: T-2 toxin; ZEN: zearalenone; DON: deoxynivalenol; OTA: ochratoxin A; NIV: nivalenol.

**Figure 2:**
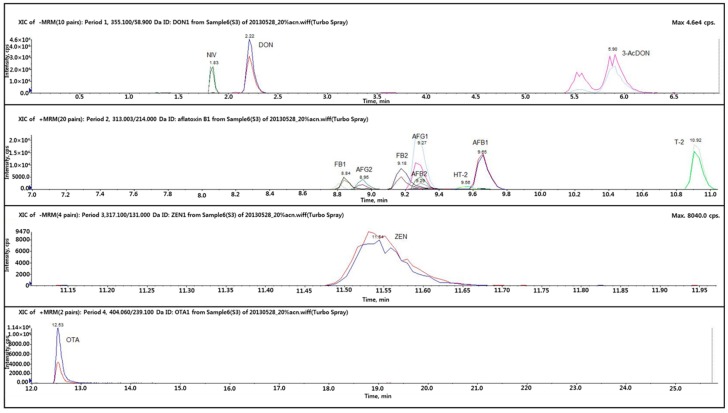
Extracted-ion chromatogram (XIC) for the mycotoxins.

Figure 1 and Figure 2 depict the total ion current chromatogram (TIC) and the extracted-ion chromatogram (XIC), respectively, for the 12 mycotoxins in the LC-MS/MS analysis. The TIC is the sum of all the ion currents in a series of mass spectra, recorded as a function of the retention time [23]. Typically, the range expands over hundreds of mass-to-charge charge units. The XIC is used to re-examine the data to detect verified analytes, to emphasize potential isomers, and to supply distinct chromatograms for compounds of interest [23]. In this study, the coefficients of correlation (R^2^) in the standard curves for AFB_1_, FB_1_, and ZEN were calculated as 1.000 in the LC-MS/MS analysis. The other toxins were analyzed with slightly lower correlation of coefficient value (R^2^) of 0.999 in their standard curves. All 12 mycotoxins could be separated on the chromatogram, and their contents were measured successfully at the optimum operation conditions for LC-MS/MS.

### 2.2. Occurrence of Mycotoxins in Raw Corn

As shown in Table 2, all the raw corn samples were contaminated at the highest levels with DON and FUMs. ZEN was also detected in most samples at a relatively high level. In most cases, very tiny amounts of AFs, NIV, derivatives of DON, OTA, HT-2, and T-2 toxin could be detected. The maximum contamination levels of DON, ZEN, and FUMs in the two samples imported from the EU were 1385.7, 79.3, and 723.8 µg/kg, respectively, and the maximum levels of those mycotoxins in the six corn samples from the US were 833.7, 200.0, and 231.6 µg/kg, respectively.

The DON levels in the corn samples did not exceed the guideline for DON (2000 µg/kg) in Korea. The average contamination levels of ZEN were 39.8 µg/kg (US) and 73.1 µg/kg (EU) (Table 2). Given the guideline for ZEN in Korea (200 µg/kg), the detected levels of ZEN in the corn samples were negligible. The average contamination levels of FUMs in the corn samples from the US and the EU were 77.2 and 473.7 µg/kg, respectively, and the maximum levels of FUMs in these samples were 231.6 and 723.8 µg/kg, respectively. All the measured contamination levels were lower than the guidelines for FUMs in Korea (Table 3).

The type of mycotoxin contamination in this study depended on the cultivation area. In a previous study, corn samples from Nigeria were highly contaminated with FUMs (10–3644 µg/kg) and AFs (0.07–109.78 µg/kg) [24]. Our results displayed a similar tendency, in that the contamination levels of FUMs were higher than those of AFs. However, interestingly, the corn samples in the previous study exhibited much lower levels of DON contamination (0.1–0.7 µg/kg) than our samples [24]. Corn samples in Switzerland were major contaminated with DON (6 of 20 samples, mean contamination level at 318 μg/kg) and FUMs (17 of 20 samples, mean contamination level at 108 μg/kg) [25]. Cereal and flour samples containing corn starch in Portugal showed the contamination with DON (2 of 18) ranging 37–111 μg/kg and only one sample was contaminated with DON, ZEN, and 15-acetyldeoxynivalenol at the same time [26]. In our study, the corn samples were contaminated with DON and FUMs at higher levels than other mycotoxins. T-2 and HT-2 could also be detected in these corn samples at high levels (263.7 and 523.3 µg/kg, respectively). In the case of corn from Brazil, FUMs (1840 µg/kg) were the most abundant contaminating mycotoxins, while DON, ZEN, and AFs were not detected [27]. As the contamination levels of mycotoxins are influenced by the geographical environment and/or other factors, constant management is necessary to obtain more generalized data.

### 2.3. Contamination Level of Mycotoxins during the Wet-Milling of Corn

#### 2.3.1. Aflatoxins

As shown in Table 4, AFs could not be measured in most corn by-product samples, except for corn steep liquor and light steep water. AFB_1_ was a contaminant in both latter sample types, at mean levels of 0.7 and 0.3 µg/kg, respectively. The relatively low contamination levels of AFs in both LSW and CSL samples can explained by the low contamination levels of AFs in raw corn itself, as shown in Table 4, and by the high carry-over ratio of AFB_1_ into cornstarch, which will be explained in the carry-over analysis (Table 8).

#### 2.3.2. Trichothecene Mycotoxins

The distribution of trichothecene mycotoxins (DON, NIV, HT-2, and T-2) is shown in Table 5. DON was detected at the highest levels among the trichothecene mycotoxins in all corn by-products. Contamination by other trichothecene mycotoxins was very low or below the LOD or LOQ in all samples. DON is found mainly in the envelope of corn and is a water-soluble toxin. Therefore, in the first stage of wet-milling, most of the DON in raw corn is transferred to LSW and CSL, which are used to produce corn gluten feed. The mean contamination levels of DON in LSW and CSL were 3641.2 and 7,417.5 µg/kg, respectively. The mean contamination level of DON in corn gluten feed was the highest (1398.8 µg/kg) among the corn by-products because the corn gluten feed was made with CSL prepared from LSW. DON was also detected in corn bran (529.8 µg/kg), corn gluten (506.2 µg/kg), and corn germ (358.9 µg/kg). Starch was low contaminated with DON.

Escobar et al. evaluated DON in wet-milled corn germ and corn oil produced in Spain [7]. DON was not detected in corn germ but was found in corn oil at 31 µg/kg. In another study performed in South Africa, DON contamination was observed in corn flour (127.8 µg/kg), corn grits (27.2 µg/kg), corn germ (215.4 µg/kg), and corn bran (941.4 µg/kg) [31]. Thus, the level of DON in corn germ was lower than ours, but that in corn bran was higher than ours. In the UK, DON contamination was identified in unprocessed corn (1750 µg/kg), corn grits (750 µg/kg), and corn animal feed (900 µg/kg) [32]. This DON contamination level in unprocessed corn was 1.5 times higher than the level we detected in raw corn from the EU.

#### 2.3.3. Fumonisins

As shown in Table 6, FUMs were mainly transferred from raw corn to corn gluten feed (2864.0 µg/kg), followed by corn gluten (2240.8 µg/kg), corn germ (1555.0 µg/kg), and corn bran (1097.7 µg/kg). No FUMs were detected in the cornstarch samples. FUMs are soluble in water; therefore, they can easily be transferred via LSW and CSL to corn gluten feed. A similar tendency was observed for DON contamination in corn gluten feed. The contamination levels of FUMs in LSW and CSL were 1738.5 and 4894.5 µg/kg, respectively.

Corn germ and corn oil produced in Spain were reported to be contaminated with FUMs at 1,303 and 27 µg/kg, respectively [7]. FUMs were also detected in corn flour (338.4 µg/kg), corn grits (61 µg/kg), corn germ (143 µg/kg), and corn bran (1157 µg/kg) collected in South Africa [31]. The contamination level of FUMs in corn bran distributed in Spain was similar to the level estimated in our study, but the level in corn germ differed greatly from ours. These mycotoxins were also detected in unprocessed corn (4000 µg/kg), corn grits (1400 µg/kg), and corn animal feed (5000 µg/kg) distributed in the UK [32]. The level of FUMs detected in unprocessed corn from the UK was much higher than the levels we detected (83.36 and 473.74 μg/kg in corn imported from the US and the EU, respectively). Interestingly, high levels of FUMs (between 2557 and 6033 µg/kg) were detected in starch produced in Italy, as well as in corn germ, bran, and grits (4569–16,008, 9713–21,244, and 586–720 µg/kg, respectively) [13]. In that study, the FUM contamination levels in the tested samples were generally higher than those in our study.

#### 2.3.4. Zearalenone and Ochratoxin A

ZEN was distributed in corn and corn by-products during wet-milling, as shown in Table 7. Due to its hydrophobicity, ZEN cannot be transferred easily from corn to LSW and CSL during the wet-milling process, so the mean contamination levels of ZEN in LSW and CSL were relatively low (2.8 and 23.8 µg/kg, respectively). The mean contamination level of ZEN was the highest in corn gluten (329.8 µg/kg) among the corn by-products, followed by corn germ (298.9 µg/kg), corn bran (142.9 µg/kg), and corn gluten feed (118.2 µg/kg). Interestingly, tiny amounts of ZEN were detected in starch (7.9 and 7.8 µg/kg). OTA existed tiny amounts in most samples, in which the contamination levels ranged from ND to 1.3 µg/kg.

Similarly, low levels of ZEN were reported for corn germ and corn oil produced by wet-milling in Spain (12 and 15 µg/kg, respectively) [7]. However, ZEN was reported to be a contaminant in corn flour (31 µg/kg), corn grits (8.6 µg/kg), corn germ (29.8 µg/kg), and corn bran (245.6 µg/kg) in South Africa [31]. The pattern of ZEN contamination in that study differed from ours. In a similar study performed in the UK, ZEN was found in unprocessed corn (350 µg/kg), corn grits (200 µg/kg), and corn animal feed (100 µg/kg) [32]. The OTA contamination levels in corn by-products have not been reported previously.

### 2.4. Distribution and Carry-Over of Mycotoxins in Corn and Corn By-Products

Generally, when corn is processed in Korea by wet-milling, the corn-derived products include 65–70% starch, 5–6% gluten, 6.5–7.5% germ, and 18–20% corn gluten feed. The amount of each corn by-product that would be yielded from 1,000 kg of corn was determined from these production levels, and the carry-over of mycotoxins into these products was thereby estimated. Tiny amounts of AFs other than AFB_1_ were detected, and their existence can be disregarded (Table 8). AFB_1_ was mainly carried over into corn gluten feed (65.0% of the total AFB_1_). OTA was distributed in cornstarch, corn gluten, and corn gluten feed. ZEN was evenly distributed in all the corn by-products except cornstarch. FUMs were mainly concentrated in corn gluten feed. Similarly, most trichothecene mycotoxins were carried over into corn gluten feed. Interestingly, NIV was mainly found in cornstarch, unlike the other trichothecene mycotoxins. Detailed data related to co-occurrence of tested mycotoxins are shown in the supplementary data (Table S1).

During the wet-milling process in Argentina, the contamination level of FUMs was higher in corn germ and bran (4210 µg/kg) than in other corn fractions—approximately 3 times higher than in raw corn (1540 µg/kg) and 13 times higher than in corn flour (358 µg/kg) [10]. In the present study, FUMs contamination was mainly found in corn gluten feed among the corn by-products, in contrast to the report from Argentina. A similar distribution study in Spain indicated that FUMs and AFs exhibited the highest distribution factors in corn gluten feed (FUMs: 317% and AFs: 288%) among the corn fractions in the dry-milling process [15]. Both of these previous studies only reported the contamination levels of mycotoxins in the corn-derived fractions produced by corn-milling, and the carry-over ratios of the tested mycotoxins into the corn-derived products could not be calculated due to the lack of production yield data in these countries.

## 3. Conclusions

Corn is a very important food and feed resource in Korea that it is mostly imported from abroad. Unfortunately, corn can easily be contaminated with fungal strains that produce mycotoxins during storage and distribution. Furthermore, mycotoxins in corn can easily be transferred to corn by-products during food processing. Therefore, the appropriate management of mycotoxin levels in corn and corn by-products is necessary to ensure food and feed safety. In this study, 12 mycotoxins were simultaneously analyzed by LC-MS/MS in corn by-products produced by wet-milling in Korea. The contamination levels of these mycotoxins were investigated after a method validation that determined the LOD values, LOQ values, and recovery ratios, according to the AOAC and CODEX Alimentarius guidelines. *Fusarium* mycotoxins such as DON, FUMs, and ZEN were the major mycotoxins in raw corn, and were mostly transferred to corn gluten feed for animals. The contamination levels of all the mycotoxins in the samples did not exceed the guidelines of the Korean Food and Drug Administration for foods. Thus, we concluded that corn and corn by-products produced by the wet-milling process in Korea can be regarded as safe from mycotoxin contamination.

## 4. Materials and Methods

### 4.1. Chemicals and Reagents

ZEN (100 µg/mL, 2 mL), T-2 toxin (100 µg/mL, 1 mL), and HT-2 toxin (100 µg/mL, 1 mL) were obtained from Sigma (St Louis, MO, USA). DON (B-trichothecenes mix 2, 100 µg/mL, 5 mL), AFs (AFs mix 1, 2 µg/mL for AFB_1_ and AFG_1_, and 0.5 µg/mL for AFB_2_ and AFG_2_, 5 mL), OTA (10 µg/mL, 5 mL), and FB_1_ and FB_2_ (FUMs mix 3, 50 µg/mL, 5 mL) were purchased from Biopure (Getzersdorf, Austria). Phosphate-buffered saline (PBS, pH 7.4) was purchased from Sigma. HPLC-grade acetonitrile and methanol were supplied by Merck (Darmstadt, Germany). Ammonium acetate (HPLC grade, 99.0%, Netherlands) and formic acid (mass spectrometry grade, 98%, St. Louis, USA) were purchased from Sigma-Aldrich (Merck KGaA, Darmstadt, Germany). For purification, a Myco6in1+ IAC column obtained from Vicam was used (Boston, MA, USA). A Myco6in1+ column is intended to simultaneous detect the multiple mycotoxins including AFs, ochratoxin, FUMs, ZEN, DON, NIV, T-2, and HT-2 toxin.

### 4.2. Samples

Cornstarch and other corn by-products were collected from corn wet-milling factories in Korea. Imported corn was used to produce cornstarch in all the factories. The 52 samples included raw corn from the US (*n* = 6) and the EU (*n* = 2), corn bran (*n* = 6), cornstarch (*n* = 8), corn gluten (*n* = 6), corn gluten feed (*n* = 6), corn germ (*n* = 6), light steep water (LSW) (*n* = 6), and corn steep liquor (CSL) (*n* = 6). All the samples were collected twice during the wet-milling process in three factories. All the corn and corn by-product samples except for LSW and CSL were packed in polyethylene bags and stored at −70 °C.

### 4.3. Sample Preparation

All the samples were milled to a particle size under 600 µm and weighed precisely to 25 g. The samples were extracted by strong shaking with 100 mL of extraction solvent (methanol:water = 70:30, *v*/*v*) for one hour. Each extract was centrifuged and filtered through filter paper (Whatman no. 4). The filtered extract (10 mL) was diluted with 90 mL of PBS, and 20 mL of the diluted extract was loaded on the IAC for purification. The IAC column was washed with PBS (10 mL) and water (10 mL). Then, the mycotoxins were eluted from the column with 5 mL of methanol at 1 drop/s. The eluted fraction was dried under nitrogen at 40 °C. After the addition of 0.5 mL of 50% methanol (containing 1% acetic acid), the mycotoxin samples were stored in vials at 4 °C until LC-MS/MS analysis.

### 4.4. Equipment

HPLC (Agilent, 1200 series, Richardson, USA)-coupled triple-quadrupole mass spectrometry (LC-MS/MS, AB, Applied Biosystems 4000, Waltham, MA, USA) with a Scherzo Sm-C18 column (2 × 150 mm, 3-µm particle size, Portland, OR, USA) was used for quantitative analysis. HPLC system were comprised of degasser, binary pump SL, autosampler, thermostatted column compartment, and thermostat (Agilent, Richardson, TX, USA). The mill (Perten, Hägersten, Sweden), homogenizer (OMNI, Kennesaw, GA, USA), shaker (Taitec, SR-2w, Saitamaken, Japan), centrifuge (Hanil Combi 514R, Gimpo, Korea), and nitration concentrator (Interface, HyperVap HV-300, Daejeon, Korea) were used in all experiments.

### 4.5. LC-MS/MS Conditions

LC was performed under gradient conditions of the mobile phase at a flow rate of 0.25 mL/min. The mobile phase consisted of solution A (20% acetonitrile with 2 mM ammonium acetate) and solution B (100% acetonitrile with 0.3% formic acid). The gradient conditions were optimized through the elevation of the percentage of solution B, as follows: 0% B from 0 to 5 min, 0–40% B from 5 to 6 min, 40% B from 6 to 8 min, 40–70% B from 8 to 9 min, 70% B from 9 to 11 min, 70–95% B from 11 to 12 min, 95% B from 12 to 16 min, 95–0% B from 16 to 17 min, and 0% B from 17 to 26 min. Ten microliters of the sample were injected into the LC-MS/MS system, and the mass spectrometry system was operated differently according to the mycotoxin. NIV, DON, and ZEN were analyzed in negative mode because of effective ionization, while the other mycotoxins were analyzed in positive mode. The conditions of the ion source in negative mode were as follows: curtain gas: 20 psig, collision gas: medium, ion spray voltage: ±4500, temperature: 500 °C, ion GS1: 50 psig, ion GS2: 50 psig, acquisition mode: MRM. The compound parameters of each ingredient are shown in Table 9.

### 4.6. Method Validation

Twelve mycotoxins were analyzed simultaneously by LC-MS/MS. An intra-laboratory validation was conducted to determine the LOD, LOQ, RSD, recovery ratio, and linearity. The LOD and the LOQ were calculated by software after repeated measurements at a signal-to-noise ratio of 3 and 10, respectively. In this study, an external standard method was applied to minimize the effects of interfering substances from the IAC pretreatment that could have interrupted the ionization in the mass spectrometer. The recovery ratios of the established methods were measured with mycotoxin-free corn as a blank sample. Individual toxins were added to the blank sample at concentrations between 12.5 and 500 μg/kg. Precision was combined with repeatability and reproducibility to measure the RSD. All the methods for determining the repeatability and reproducibility followed the guidelines of the AOAC [20].

### 4.7. Wet-Milling Process of Corn

**Figure 3:**
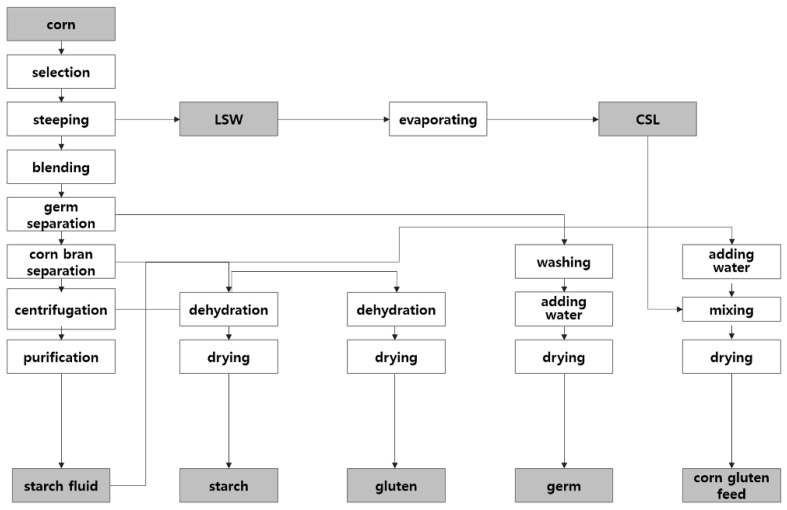
Wet-milling process to produce cornstarch and corn by-products.

Cornstarch exists on the outside of the endosperm, while gluten is located on the inside. Other parts of corn (e.g., the germ) are the main source for the production of corn oil. In general, corn contains 13.5% moisture, 70% carbohydrate, 8.5% protein, 4.5% fat, 2% fiber, and 1.5% ash. Corn is separated into starch, gluten, germ, and corn gluten feed during the wet-milling process, with yields of 70, 4, 6–7, and 17%, respectively [13,33]. Light steep water (LSW) and Corn steep liquor (CSL) are intermediate by-products that contain soluble proteins, lactic acid, sugars, vitamins, and minerals. CSL is sprinkled on the corn bran and dried to produce corn gluten feed for animals [16]. The wet-milling process is shown in Figure 3.

## Figures and Tables

**Table 1:** Summary of the method validation

Mycotoxin ^a^	AFB_1_	AFB_2_	AFG_1_	AFG_2_	FB_1_	FB_2_	HT-2	T-2	ZEN	DON	OTA	NIV
Coefficient of correlation (R^2^)	1.000	0.999	0.999	0.999	1.000	0.999	0.999	0.999	1.000	0.999	0.999	0.999
Range (μg/kg)	0.25–50.5	0.06–12.4	0.25–50.3	0.06–12.4	2.5–500	2.5–500	0.25–50	0.25–50	0.25–50	5–1000	0.25–50.5	5–1000
Spiked level (μg/kg)	12.5	12.5	12.5	12.5	500.0	500.0	500.0	500.0	100.0	250.0	25.0	250.0
Recovery (%)	87.7	99.7	86.9	112.1	101.0	81.9	72.7	82.5	93.5	108.8	103.1	128.9
RSD (%) *	7.4	10.2	6.5	13.8	11.5	14.5	13.6	4.1	3.8	4.7	4.3	7.8
LOD (μg/kg)	0.1	0.3	0.3	0.5	2.4	2.3	1.7	0.1	0.2	4.1	0.1	4.8
LOQ (μg/kg)	0.4	0.9	1.0	1.6	8.2	7.8	5.6	0.3	0.6	13.6	0.3	16.0

^a^ AFB_1_, AFB_2_, AFG_1_, AFG_2_: aflatoxins B_1_, B_2_, G_1_, and G_2_; FB_1_, FB_2_: fumonisins B_1_ and B_2_; HT-2: HT-2 toxin; T-2: T-2 toxin; ZEN: zearalenone; DON: deoxynivalenol; OTA: ochratoxin A; NIV: nivalenol. RSD: relative standard deviation; LOD: limit of detection; LOQ: limit of quantification. * This value was calculated from 7 replicates of mycotoxins analysis.

**Table 2:** Mycotoxin occurrence in raw corn

Toxin ^a^	Corn (US, *n* = 6) (EU, *n* = 2)	Mean ± SD (µg/kg)	Range (µg/kg)	Contamination Rate (%)
**AFB_1_**	US	0.1 ± 0.2	ND–0.4	33.3
	EU	ND	ND	0
**AFB_2_**	US	ND	ND	0
	EU	ND	ND	0
**AFG_1_**	US	ND	ND	0
	EU	ND	ND	0
**AFG_2_**	US	ND	ND	0
	EU	ND	ND	0
**DON**	US	213.6 ± 320.4	LOD–833.7	100
	EU	1142.5 ± 343.9	899.3–1385.7	100
**NIV**	US	<LOQ	<LOQ	33.3
	EU	ND	ND	0
**HT-2**	US	<LOQ	<LOQ	33.3
	EU	ND	ND	0
**T-2**	US	2.0 ± 4.1	10.3	33.3
	EU	ND	ND	0
**FB_1_**	US	61.4 ± 87.3	LOD–177.7	100
	EU	363.9 ± 255.9	183.0–544.8	100
**FB_2_**	US	15.8 ± 24.9	LOD–53.9	100
	EU	109.8 ± 97.8	40.6–179.0	100
**ZEN**	US	39.8 ± 79.7	ND–200.0	83.3
	EU	73.1 ± 8.7	67.0–79.3	100
**OTA**	US	ND	<LOQ	0
	EU	ND	<LOQ	0

^a^ AFB_1_, AFB_2_, AFG_1_, AFG_2_: aflatoxins B_1_, B_2_, G_1_, and G_2_; FB_1_, FB_2_: fumonisins B_1_ and B_2_; HT-2: HT-2 toxin; T-2: T-2 toxin; ZEN: zearalenone; DON: deoxynivalenol; OTA: ochratoxin A; NIV: nivalenol. LOD: limit of detection; LOQ: limit of quantification; ND: not detected.

**Table 3:** Guidance values for mycotoxins in foodstuffs in US, EU, and Korea

Mycotoxin	Products	Guidance Value in μg/kg (ppb)		
		US [28]	EU [29]	Korea [30]
**Aflatoxins** **(B_1_ + B_2_ + G_1_ + G_2_)**	Cereals and cereal products (simple processing; cutting and crushing, etc.)	–	5.0–10.0	15.0 (10.0 for AFB_1_)
	Maize and rice to be subjected to sorting or other physical treatment before human consumption or use as an ingredient in foodstuffs	–	10.0	15.0 (10.0 for AFB_1_)
	Processed cereal-based foods and baby foods for infants and young children	–	0.10	0.10 (0.10 for AFB_1_)
	Foods	20	–	–
**Ochratoxin A**	Unprocessed cereals	–	5.0	5.0
	Cereal products (simple processing; cutting and crushing, etc.)	–	3.0	5.0
	Processed cereal-based foods and baby foods for infants and young children	–	0.50	0.50
**Fumonisins** **(B_1_ + B_2_ + B_3_)**	Unprocessed corn	–	4000	4000
	Corn products (simple processing; cutting and crushing, etc.)	–	1000	2000
	Corn products and cereal (containing more than 50% of simple processing)	–	–	1000
	Corn products for popcorn	3000	–	1000
	Corn products (dry milled)	4000	–	–
	Corn products for masa production	4000	–	–
	Breakfast cereals and snacks (corn based)	–	800	1000
	Processed corn-based foods and baby foods for infants and young children	–	200	–
**Deoxynivalenol**	Unprocessed corn	–	1750	2000
	Corn products (simple processing; cutting and crushing, etc.)	–	–	2000
	Processed corn-based foods and baby foods for infants and young children	–	200	0.2
**Zeralenone**	Unprocessed cereals	–	100	200
	Cereal products (simple processing; cutting and crushing, etc.)	–	–	200
	Unprocessed corn	–	350	–
	Refined corn oil	–	400	–
	Breakfast cereals and snacks (corn based)	–	100	50
	Processed corn-based foods for infants and young children	–	20	20

**Table 4:** Aflatoxins in corn by-products produced by wet-milling

Sample Type ^a^	AFB_1_		AFB_2_		AFG_1_		AFG_2_	
	Mean ± SD (μg/kg)	Range (μg/kg)	Mean ± SD (μg/kg)	Range (μg/kg)	Mean ± SD (μg/kg)	Range (μg/kg)	Mean ± SD (μg/kg)	Range (μg/kg)
**Corn gluten (*n* = 6)**	0.2 ± 0.3	LOD–1.0	ND	ND	ND	ND	ND	ND
**Starch (corn from US, *n* = 6)**	<LOQ	<LOQ	ND	ND	ND	ND	ND	ND
**starch (corn from EU, *n* = 2)**	ND	ND	ND	ND	ND	ND	ND	ND
**Corn gluten feed (*n* = 6)**	0.2 ± 0.3	LOD–0.7	ND	ND	ND	ND	ND	ND
**Corn germ (*n* = 6)**	0.2 ± 0.3	LOD–0.6	ND	ND	ND	ND	ND	ND
**Corn bran (*n* = 6)**	0.2 ± 0.3	LOD–0.5	ND	ND	ND	ND	ND	ND
**Light steep water (*n* = 6)**	0.3 ± 0.8	ND–0.8	ND	ND	ND	ND	ND	ND
**Corn steep liquor (*n* = 6)**	0.7 ± 1.0	LOD–2.0	ND	ND	ND	ND	ND	ND

^a^ AFB_1_, AFB_2_, AFG_1_, AFG_2_: aflatoxins B_1_, B_2_, G_1_, and G_2_. LOD: limit of detection; LOQ: limit of quantification; ND: not detected.

**Table 5:** Trichothecene mycotoxins in corn by-products produced by wet-milling

**Sample Type ^a^**	**DON**		**NIV**	
	**Mean ± SD (µg/kg)**	**Range (µg/kg)**	**Mean ± SD (µg/kg)**	**Range (µg/kg)**
**Corn gluten (*n* = 6)**	506.2 ± 210.6	129.0–702.3	<LOQ	<LOQ
**Starch (corn from US, *n* = 6)**	2.9 ± 7.0	ND–17.2	4.0 ± 9.9	ND–24.3
**Starch (corn from EU, *n* = 2)**	<LOQ	<LOQ	ND	ND
**Corn gluten feed (*n* = 6)**	1398.8 ± 884.1	49.0–2,490.0	4.5 ± 10.9	ND–26.8
**Corn germ (*n* = 6)**	358.9 ± 217.4	134.0–647.0	<LOQ	<LOQ
**Corn bran (*n* = 6)**	529.8 ± 313.1	118.8–952.0	3.8 ± 9.3	ND–22.8
**Light steep water (*n* = 6)**	3641.2 ± 2075.3	64.4–6340.0	25.6 ± 42.7	LOD–101.75
**Corn steep liquor (*n* = 6)**	7417.5 ± 4409.4	992.5–11,600.0	41.3 ± 64.0	ND–129.3
**Sample Type ^a^**	**T-2**		**HT-2**	
	**Mean ± SD (µg/kg)**	**Range (µg/kg)**	**Mean ± SD (µg/kg)**	**Range (µg/kg)**
**Corn gluten (*n* = 6)**	12.2 ± 10.3	LOD–24.8	15.4 ± 10.3	LOD–28.5
**Starch (corn from US, *n* = 6)**	0.2 ± 0.3	ND–0.8	<LOQ	<LOQ
**Starch (corn from EU, *n* = 2)**	ND	ND	ND	ND
**Corn gluten feed (*n* = 6)**	8.3 ± 8.3	ND–21.1	23.6 ± 15.1	LOD–39.7
**Corn germ (*n* = 6)**	6.9 ± 7.6	ND–18.0	1.0 ± 2.5	ND–6.1
**Corn bran (*n* = 6)**	3.2 ± 5.0	ND–10.8	3.9 ± 4.4	ND–9.5
**Light steep water (*n* = 6)**	5.7 ± 4.4	8.3–8.8	11.3 ± 3.8	7.8–18.6
**Corn steep liquor (*n* = 6)**	9.9 ± 8.1	ND–18.2	33.2 ± 18.3	13.0–57.3

^a^ DON: deoxynivalenol; NIV: nivalenol; HT-2: HT-2 toxin; T-2: T-2 toxin. LOD: limit of detection; LOQ: limit of quantification; ND: not detected.

**Table 6:** Fumonisins in corn by-products produced by wet-milling

Sample Type ^a^	FB_1_		FB_2_	
	Mean ± SD (µg/kg)	Range (µg/kg)	Mean ± SD (µg/kg)	Range (µg/kg)
**Corn gluten (*n* = 6)**	1415.9 ± 818.3	167.9–2641.0	824.9 ± 424.6	53.6–1166.7
**Starch (corn from US, *n* = 6)**	6.8 ± 5.7	ND–13.6	3.0 ± 4.6	ND–8.9
**Starch (corn from EU, *n* = 2)**	<LOQ	<LOQ	<LOQ	<LOQ
**Corn gluten feed (*n* = 6)**	2520.0 ± 1,703.9	133.2–4835.0	344.0 ± 237.7	18.9–735.5
**Corn germ (*n* = 6)**	1042.9 ± 817.1	211.5–2220.0	512.1 ± 498.0	72.0–1237.5
**Corn bran (*n* = 6)**	820.3 ± 633.1	138.7–1763.3	277.4 ± 245.2	54.2–669.0
**Light steep water (*n* = 6)**	1585.3 ± 979.0	279.4–1847.5	153.2 ± 110.4	29.7–355.5
**Corn steep liquor (*n* = 6)**	4594.6 ± 3262.6	760.0–8275.0	299.9 ± 220.1	46.1–519.3

^a^ FB_1_, FB_2_: fumonisins B_1_ and B_2_; LOQ: limit of quantification; ND: not detected.

**Table 7:** Zearalenone and ochratoxin A in corn by-products produced by wet-milling

Sample Type ^a^	ZEN		OTA	
	Mean ± SD (µg/kg)	Range (µg/kg)	Mean ± SD (µg/kg)	Range (µg/kg)
**Corn gluten (*n* = 6)**	329.8 ± 237.6	15.2–604.0	0.5 ± 0.4	LOD–1.0
**Starch (corn from US, *n* = 6)**	7.9 ± 9.2	ND–20.5	0.1 ± 0.1	<LOQ
**Starch (corn from EU, *n* = 2)**	7.8 ± 0.0	7.8–7.8	<LOQ	<LOQ
**Corn gluten feed (*n* = 6)**	118.2 ± 84.6	2.2–199.7	0.1 ± 0.2	ND–0.4
**Corn germ (*n* = 6)**	298.9 ± 245.0	26.4–610.3	0.2 ± 0.2	ND–0.5
**Corn bran (*n* = 6)**	142.9 ± 140.6	11.3–317.3	0.2 ± 0.3	ND–0.6
**Light steep water (*n* = 6)**	2.8 ± 3.6	LOD–9.0	1.0 ± 0.9	0.4–2.2
**Corn steep liquor (*n* = 6)**	23.8 ± 22.6	0.6–55.0	0.8 ± 0.4	0.4–1.3

^a^ ZEN: zearalenone; OTA: Ochratoxin A; LOQ: limit of quantification.

**Table 8:** Distribution and carry-over of mycotoxins in corn by-products

Toxin ^a^	Cornstarch (µg *, % ^†^)	Corn Gluten (µg *, % ^†^)	Corn Gluten Feed (µg *, % ^†^)	Corn Germ (µg*, % ^†^)	Total (µg)
**AFB_1_**	ND	9.9 (15.1%)	42.5 (65.0%)	13.1 (19.9%)	65.4
**OTA**	30.4 (32.7%)	28.4 (30.6%)	23.2 (25.0%)	10.9 (11.7%)	92.9
**ZEN**	5343.0 (7.8%)	19,119.4 (27.7%)	22,832.9 (33.1%)	21,660.9 (31.4%)	68,956.1
**FB_1_**	3449.3 (0.5%)	82,082.9 (12.7%)	486,950.7 (75.1%)	75,575.4 (11.7%)	648,058.3
**FB_2_**	1501.4 (0.9%)	47,822.0 (31.3%)	66,481.2 (43.5%)	37,109.4 (24.3%)	152,914.1
**DON**	1450.7 (0.4%)	29,343.2 (9.0%)	270,305.3 (82.6%)	26,004.3 (8.0%)	327,103.6
**NIV**	2049.3 (64.3%)	ND	863.8 (27.1%)	275.4 (8.6%)	3,188.4
**T-2**	96.4 (3.3%)	706.1 (24.2%)	1607.7 (55.2%)	502.2 (17.3%)	2,912.4
**HT-2**	ND	889.9 (16.1%)	4552.7 (82.5%)	73.9 (1.4%)	5516.4

* The total levels of mycotoxins in corn by-products produced from one ton of raw corn ^†^ Percent distribution of mycotoxins in corn by-products produced from one ton of raw corn. ^a^ AFB_1_: aflatoxin B_1_; OTA: ochratoxin A; ZEN: zearalenone; FB_1_, FB_2_: fumonisins B_1_ and B_2_; DON: deoxynivalenol; NIV: nivalenol; HT-2: HT-2 toxin; T-2: T-2 toxin. ND: not detected.

**Table 9:** LC-MS/MS parameters of mycotoxins under optimized conditions

ID ^a^ (Toxins)	Type	Q1 (Precursor Ion)	Q3 (Product Ion)	TIME (Ms)	DP (Volts)	EP (Volts)	CE (Volts)	CXP (Volts)
**NIV**	[M + CH_3_COO^−^]^−^	371.1	281.1	150	−15	−3.5	−18	−2
		371.1	59.0	150	−15	−3.5	−38	−6
**DON**	[M + CH_3_COO^−^]^−^	355.1	59.0	150	−15	−3.5	−40	−6
		355.1	295.2	150	−15	−3.5	−14	−2
**AFB_1_**	[M + H]^+^	313.0	241.0	80	66	10	43	4
		313.0	285.3	80	66	10	43	4
**AFB_2_**	[M + H]^+^	315.0	286.9	80	66	10.5	39	6
		315.0	259.0	80	66	10.5	33	4
**AFG_1_**	[M + H]^+^	329.0	243.0	80	61	11	37	6
		329.0	200.0	80	61	11	55	6
**AFG_2_**	[M + H]^+^	331.0	313.1	80	61	10.5	39	8
		331.0	245.3	80	61	10.5	39	8
**FB_1_**	[M + H]^+^	722.4	334.3	80	76	10.5	51	6
		722.4	352.3	80	76	10.5	47	6
**FB_2_**	[M + H]^+^	706.4	336.3	80	76	10	51	6
		706.4	318.3	80	76	10	51	6
**HT−2**	[M + NH_4_]^+^	442.2	263.2	80	50	10	19	6
		442.2	215.2	80	50	10	19	6
**T−2**	[M + NH_4_]^+^	484.3	305.1	80	50	10	19	6
		484.3	215.2	80	50	10	23	6
**ZEN**	[M − H]^−^	317.1	131.0	100	−50	−4.5	−40	−2
		317.1	175.0	100	−50	−4.5	−34	−2
**OTA**	[M + H]^+^	404.1	239.1	300	36	8.5	31	6
		404.1	357.9	300	36	8.5	21	6

^a^ AFB_1_, AFB_2_, AFG_1_, AFG_2_: aflatoxins B_1_, B_2_, G_1_, and G_2_; FB_1_, FB_2_: fumonisins B_1_ and B_2_; HT-2: HT-2 toxin; T-2: T-2 toxin; ZEN: zearalenone; DON: deoxynivalenol; OTA: ochratoxin A; NIV: nivalenol. DP: declustering potential; EP: entrance potential; CE: collision energy; CXP: collision cell exit potential.

## Supplementary Materials

The following are available online at http://www.mdpi.com/2072-6651/10/8/319/s1, Table S1: Co-occurrence of mycotoxins in all samples.
