# Supplementary material for: Distribution Analysis of Twelve Mycotoxins in Corn and Corn-Derived Products by LC-MS/MS to Evaluate the Carry-Over Ratio during Wet-Milling
Source: Toxins (Basel). 2018 Aug 6;10(8):319. doi: 10.3390/toxins10080319 (PMC6115783; doi:10.3390/toxins10080319)
Supplement: Supplementary file 1 [file toxins-10-00319-s001.pdf]

Supplementary Materials: Distribution Analysis of Twelve Mycotoxins in Corn and Corn-Derived Products by LC-MS/MS to Evaluate the Carry-Over Ratio during Wet-Milling

Juhee Park, Dong-Ho Kim, Ji-Young Moon, Jin-Ah An, Young-Woo Kim, Soo-Hyun Chung and Chan Lee

Table S1. Co-occurrence of mycotoxins in all samples.

	No.	AFB$_1$	AFB$_2$	AFG$_1$	AFG$_2$	FB$_1$	FB$_2$	HT-2	T-2	ZEN	DON	OTA	NIV
Corn (US)	1	-	-	-	-	+	+	-	-	++	++	-	-
	2	-	-	-	-	+	+	-	-	-	++	-	-
	3	+	-	-	-	+	+	+	-	++	+	-	+
	4	-	-	-	-	++	++	-	++	++	++	-	-
	5	++	-	-	-	++	+	+	-	++	++	-	+
	6	-	-	-	-	++	++	-	++	++	++	-	-
Corn (EU)	1	-	-	-	-	++	++	-	-	++	++	-	-
	2	-	-	-	-	++	++	-	-	++	++	-	-
Corn starch (US)	1	-	-	-	-	-	-	-	-	++	-	-	-
	2	-	-	-	-	+	+	-	-	-	-	-	-
	3	+	-	-	-	++	+	+	-	++	++	+	++
	4	-	-	-	-	++	++	-	++	++	+	++	-
	5	+	-	-	-	++	+	+	-	++	+	-	+
	6	-	-	-	-	++	++	-	++	++	+	-	-
Corn starch (EU)	1	-	-	-	-	+	+	-	-	++	-	+	-
	2	-	-	-	-	+	+	-	-	++	-	-	-
Corn gluten	1	+	-	-	-	++	++	-	++	++	++	+	-
	2	-	-	-	-	++	++	+	++	++	++	-	-
	3	++	-	-	-	++	++	+	-	++	++	+	+
	4	-	-	-	-	++	++	+	++	++	++	++	-
	5	++	-	-	-	++	++	+	-	++	++	+	+
	6	-	-	-	-	++	++	++	++	++	++	++	-
Corn gluten feed	1	++	-	-	-	++	++	++	++	++	++	+	-
	2	-	-	-	-	++	++	++	++	++	++	-	-
	3	++	-	-	-	++	++	+	-	++	++	+	-
	4	-	-	-	-	++	++	++	++	++	++	++	-
	5	++	-	-	-	++	++	++	++	++	++	+	++
	6	-	-	-	-	++	++	++	++	++	++	++	-
Corn germ	1	+	-	-	-	++	++	-	++	++	++	+	-
	2	-	-	-	-	++	++	+	++	++	++	-	-
	3	++	-	-	-	++	++	+	-	++	++	+	+
	4	-	-	-	-	++	++	+	++	++	++	++	-
	5	++	-	-	-	++	++	+	-	++	++	+	+
	6	-	-	-	-	++	++	++	++	++	++	++	-
Corn bran	1	+	-	-	-	++	++	+	-	++	++	+	-
	2	-	-	-	-	++	++	-	+	++	++	-	-
	3	++	-	-	-	++	++	+	-	++	++	+	++
	4	-	-	-	-	++	++	++	++	++	++	++	-
	5	++	-	-	-	++	++	++	-	++	++	+	+
	6	-	-	-	-	++	++	++	++	++	++	++	-

	**1**	-	-	-	-	++	++	++	++	++	++	++	++
	**2**	-	-	-	-	++	++	++	++	++	++	++	-
**LSW**	**3**	-	-	-	-	++	++	++	-	+	++	++	+
	**4**	-	-	-	-	++	++	++	++	+	++	-	-
	**5**	++	-	-	-	++	++	++	-	++	++	++	++
	**6**	-	-	-	-	++	++	++	++	++	++	++	-
	**1**	-	-	-	-	++	++	++	++	++	++	++	-
	**2**	-	-	-	-	++	++	++	++	++	++	++	-
**CSL**	**3**	++	-	-	-	++	++	++	-	++	++	++	++
	**4**	-	-	-	-	++	++	++	++	++	++	++	-
	**5**	++	-	-	-	++	++	++	-	++	++	++	++
	**6**	-	-	-	-	++	++	++	++	++	++	++	-

-: not detected; +: between LOD and LOQ; ++: > LOQ.
